# Online Spatial Normalization for Real-Time fMRI

**DOI:** 10.1371/journal.pone.0103302

**Published:** 2014-07-22

**Authors:** Xiaofei Li, Li Yao, Qing Ye, Xiaojie Zhao

**Affiliations:** 1 College of Information Science and Technology, Beijing Normal University, Beijing, China; 2 State Key Laboratory of Cognitive Neuroscience and Learning, Beijing Normal University, Beijing, China; Yale University, United States of America

## Abstract

Real-time functional magnetic resonance imaging (rtfMRI) is a recently emerged technique that demands fast data processing within a single repetition time (TR), such as a TR of 2 seconds. Data preprocessing in rtfMRI has rarely involved spatial normalization, which can not be accomplished in a short time period. However, spatial normalization may be critical for accurate functional localization in a stereotactic space and is an essential procedure for some emerging applications of rtfMRI. In this study, we introduced an online spatial normalization method that adopts a novel affine registration (AFR) procedure based on principal axes registration (PA) and Gauss-Newton optimization (GN) using the self-adaptive β parameter, termed PA-GN(β) AFR and nonlinear registration (NLR) based on discrete cosine transform (DCT). In AFR, PA provides an appropriate initial estimate of GN to induce the rapid convergence of GN. In addition, the β parameter, which relies on the change rate of cost function, is employed to self-adaptively adjust the iteration step of GN. The accuracy and performance of PA-GN(β) AFR were confirmed using both simulation and real data and compared with the traditional AFR. The appropriate cutoff frequency of the DCT basis function in NLR was determined to balance the accuracy and calculation load of the online spatial normalization. Finally, the validity of the online spatial normalization method was further demonstrated by brain activation in the rtfMRI data.

## Introduction

Real-time functional magnetic resonance imaging (rtfMRI) is a recently emerged technique that permits the online observation of brain activity during recording. This technique is essential for a variety of applications, such as neurofeedback, in which subjects are trained to self-regulate the local blood oxygen level dependent (BOLD) response in specific brain areas to improve their behavioral performance [1], [2]. Such types of applications demand that the image data is acquired in each repetition time (TR), and preprocessing and statistical analysis should be promptly performed in a short time period, which is usually shorter than a single TR. In many rtfMRI softwares, such as Turbo-BrainVoyager (TBV, http://www.brainvoyager.com/) and the real-time process modules of Analysis of Functional NeuroImages (AFNI, http://afni.nimh.nih.gov/) [3], the real-time preprocessing primarily consists of head motion correction [4], [5] and spatial smoothing [6], [7], while spatial normalization is an optional preprocessing procedure that alleviates the inter-individual anatomical variance by normalizing the individual image into a stereotactic space [8], [9], such as Talairach space [10], [11] and Montreal Neurological Institute (MNI) space [12].

In offline fMRI applications, spatial normalization is usually used as a standard procedure when an accurate identification of specific functional regions is needed. In many rtfMRI applications, such as the clinical brain operation [13], [14], [15], the accurate functional localization of brain regions is also an indispensable pre-requisite condition for reducing subjectivity. Online spatial normalization provides an accurate position from the stereotactic atlas, such as Automated Anatomical Labeling (AAL) [16] and Brodmann’s Area (BA), in rtfMRI localization, even if the disabled patients are unable to perform the localization task [17]. Recently, application of the rtfMRI technique on the self-regulation of brain connectivity and network activities has become an attractive topic [18], [19], [20], [21]. In these applications, spatial normalization may be an essential procedure prior to network analysis, such as semi-blind independent component analysis (ICA) [22], [23], when template masks are used or when the image needs to be analyzed in a stereotactic space. For example, the default mode network (DMN) [24] of the individual may be automatically measured using a DMN template from MNI space [22].

Some commomly used offline spatial normalization methods generally cannot be accomplished in a single TR on a current typical personal computer. One such method [3] registers the image into Talairach space by manually identifying the anterior commissure (AC) and posterior commissure (PC) to acquire the normalization parameters, which limits the reliability of these methods [8] and its application in rtfMRI. Another type of method aims at minimizing a cost function which could be least squares, mutual information and so on, such as the spatial mornalization in SPM (http://www.fil.ion.ucl.ac.uk/spm/), 3dAllineate and 3dQwarp in AFNI. These methods automatically registers the image to a stereotactic space using a non-rigid transformation that normally requires a very time-consuming iterative registration process that is longer than one TR to estimate the optimal normalization parameters [8]. Using the method in SPM, the source image is often selected as the mean image over all of the realigned images, which also hinders its application in rtfMRI.

To take advantage of regional localization in stereotactic space, a few studies have attempted to apply spatial normalization in rtfMRI [25], [26]. One method is derived from SPM99, which has been integrated into the TurboFIRE software [27]. This method uses a preparatory run to estimate the normalization parameters offline, generates a lookup table to map the coordinates in the source image space to MNI space, and acquires the Talairach coordinates in subsequent online runs [26]. The second method is based on SPM2, which is used online to measure and display BOLD signal changes from the user-selected areas labeled with AAL and BA nomenclature [25]. Specifically, the normalization parameters by which the individual’s images are fitted to the anatomical templates of AAL and BA are estimated offline from a preparatory run and are used in images transformed in the following online runs. In these methods, the image registration determines the normalization parameters offline in a preparatory run. However, the inter-run motion gradually accumulates and may grow quite large in the subsequent runs; even the sudden large head motions may occur in an on-going run or during the interval between runs. These factors can result in an underestimation of image registraion [5] and make the previous normalization parameters unsuitable for the images in subsequent online runs. In addition, the magnetic field inhomogeneities change over time in fMRI, which result in shape distortions in the brain images that cannot be corrected using a rigid body transformation [28], [29].

Based on these findings, we advanced the offline spatial normalization method in SPM8 and implemented an online spatial normalization method that can be accomplished in a time interval shorter than a TR (such as 2 s). This method consists of procedures of affine registration (AFR) and nonlinear registration (NLR) [30], which is based on discrete cosine transform (DCT). First, we proposed an AFR method based on principal axes registration (PA) [31], [32], [33] and Gauss-Newton optimization (GN) [30] using a self-adaptive β parameter, termed as PA-GN(β) AFR. Second, we verified PA-GN(β) AFR using both simulation and real data, and the results were compared with those obtained using traditional AFR. Third, to balance the accuracy and runtime of our spatial normalization method, an appropriate cutoff frequency of DCT basis function in NLR was selected using the bisection method. Finally, we applied the online spatial normalization to rtfMRI data from a finger-tapping experiment to further validate its efficacy in brain activation analysis. These results were also compared with those obtained using the offline spatial normalization.

## Materials and Methods

### Data Acquisition

Real data were acquired from a finger tapping run in an rtfMRI experiment, which consisted of eight on-going runs [34]. Twenty volunteers (age 22.3±1.6, 8 females) participated in the experiment, which was approved by the Institutional Review Board (IRB) of the State Key Laboratory of Cognitive Neuroscience and Learning in Beijing Normal University; all of the subjects signed informed consent prior to scanning. The data are available from the Dryad Digital Repository: http://dx.doi.org/10.5061/dryad.1642b. The run, which lasted 4.5 min, consisted of five rest blocks and four task blocks, of which each block lasted 30 s. During the rest blocks, a text cue “REST” was shown in the center of the screen and the subjects were instructed to take a rest. In the task blocks, a text cue “PUSH” was shown, and the subjects were instructed to tap their right-hand fingers. In addition, the subjects were instructed to hold their heads as still as possible and keep their eyes open during the entire run.

Brain scans was performed at the MRI Center of Beijing Normal University using a 3.0-T Siemens MRI scanner. A single-shot T2*-weighted gradient-echo, echo-planar imaging (EPI) sequence (TR/TE/flip angle = 2000 ms/40 ms/90°, matrix size = 64×64, voxel size = 3.1×3.1×4.8 mm^3^, slice thickness = 4 mm, slice gap = 0.8 mm) was used to acquire each image with 32 axial slices in the interleaved order. The subjects’ heads were cushioned to reduce their head movements.

### PA-GN(β) Affine Registration

**Figure 1:**
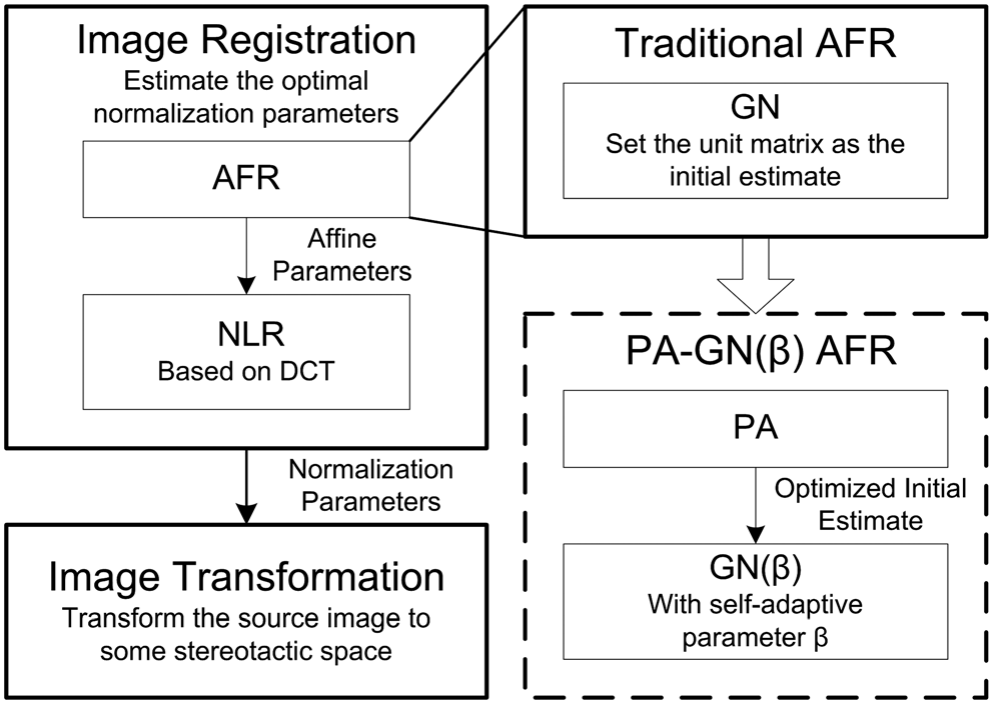
The workflow of spatial normalization (left) and advanced affine registration (right). The image registration includes affine registration (AFR) and nonlinear registration (NLR), which are used to estimate the optimal normalization parameters. The image transformation is then used to transform the source images to a stereotactic space using the estimated parameters; tri-linear interpolation is used in this study. The PA-GN(β) AFR method is advanced based on traditional AFR, in which the PA provides a better initial estimate for GN with the self-adaptive β parameter for the iteration step adjustment.



Affine registration (AFR) is normally the first step of spatial normalization (Figure 1), in which the optimal twelve-parameter vector **q** derived from the affine transformation matrix **M** is estimated to fit the source image (**f**) to the reference image (**g**). The Gauss-Newton optimization (GN) algorithm is used to minimize the cost function (CF), which is selected as the mean squared differences between images **f** and **g** [8]. The iterative GN procedure starts with the initial estimate **q** ^(0)^; at the *n*th iteration, the value of **q** ^(*n*)^ is updated according to the following rule [35]:

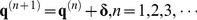
(1)where **δ** is the iteration step.

#### Initial Estimate Provided by Principle Axes Registration

In spatial normalization, the AFR from a random individual image space to a standard stereotactic space is relatively complex; this makes GN a tedious iterative process, particularly when meeting an inappropriate initial estimate [33]. In traditional AFR, the initial estimate for GN is extracted from the unit matrix, which does not provide any prior information for registration. Here, using the coarse-to-fine model, the principal axes registration (PA) [33], which is fast and simple, is used to provide a coarse but more appropriate initial estimate for GN than the unit matrix in the traditional AFR (Figure 1).

At first, images **f** and **g** are downsampled by 50% to reduce the amount of data and save time. For image **f**, the mass of the voxel *i* with the coordinates **u** *_i_* = (*x_i_*, *y_i_*, *z_i_*) is f(**u** *_i_*). The centroid of image **f**, **C**_f_ = (*x_c_*, *y_c_*, *z_c_*), is then calculated using Equation (2) [32], and the inertia matrix **I**_f_ is calculated using Equation (3a) [31].
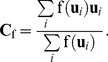
(2)

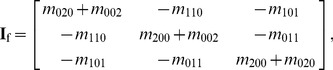
(3a)


where



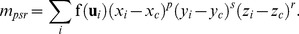
(3b)


Three eigenvectors of **I**_f_ are the principal axes: **e** *_x_* = [*e*_11_ *e*_21_ *e*_31_]^T^, **e** *_y_* = [*e*_12_ *e*_22_ *e*_32_]^T^, **e** *_z_* = [*e*_13_ *e*_23_ *e*_33_]^T^; these axes lie closest to the *x*-axis, *y*-axis, and *z*-axis of the Cartesian coordinate system and point in the respective positive direction of the corresponding axis. Next, the eigenvector matrix **E**_f_ = [**e** *_x_* **e** *_y_* **e** *_z_*] is obtained [31]. Similarly, the centroid **C**_g_ and eigenvector matrix **E**_g_ of image **g**, where **v** *_i_* is the coordinate of voxel *i*, are calculated and saved to avoid a repeated calculation due to duplicate templates in the spatial normalization. To align the image **f** to image **g**, the PA is used to superpose the centroids and principal axes of images **f** and **g**, which can be defined using the following equation [32]:
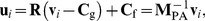
(4a)

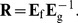
(4b)


The parameter vector derived from the matrix **M**_PA_ is the optimized initial estimate, **q** ^(0)^, for GN.

#### Iteration Step Adjustment using the Self-adaptive β Parameter

In addition to the initial estimate, the iteration step has a large effect on the convergence speed of GN. In a previous GN(α) method [35], the α parameter, which allows the adaptive change of the iteration step, is adopted to make the GN converge more rapidly. However, the linear search method used to determine the α parameter that fits the Wolfe conditions [36] is relatively complex and time-consuming. To meet the real-time requirement of rtfMRI and improve the GN performance, a newly defined β parameter, which also meets the Wolfe conditions, is introduced to self-adaptively adjust the iteration step **δ** in Equation (1) as follows:
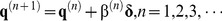
(5)where β^(*n*)^ is β at the *n*th iteration (Figure 1). Here, the iterative procedure is repeated until the change rate Δ^(*n*)^ of the cost function CF^(*n*)^ reaches the following condition:



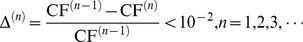
(6)The iteration step **δ** is closely linked to Δ^(*n*)^. If the cost function changes rapidly (e.g., at the beginning of the iteration), **δ** could be increased, and vice-versa (e.g., near the end of the iteration). The β parameter related to Δ^(*n*)^ is defined as follows:
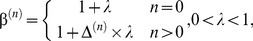
(7)where *λ* is the constraint factor that prevents excessive increments in the step. If *λ* is more than 1, then β^(*n*)^ might be undesirable for the Wolfe conditions. To ensure that β^(*n*)^ adaptively adjusts **δ** and keeps the iteration process stable, *λ* should meet the following criteria: (1) the average number of iterations is as small as possible and (2) the CF change curves and their iterations are smooth. Here, it is recommended that *λ* be set to 0.5 according to the experimental results.

### Nonlinear Registration with Appropriate Cutoff Frequency

Following AFR, the nonlinear registration (NLR) procedure is often used to reduce the gross shape differences that are not accounted for by linear deformation (Figure 1). Here, the NLR method is modeled by the combination of a series of discrete cosine transform (DCT) basis functions, and the GN algorithm is used to determine the optimal coefficients describing the nonlinear deformations [30]. To save time in the context of maintaining accuracy, the iteration stops when the change rate of CF is less than 10^−2^ same as the condition of Equation (6)

The number *N* of the coefficients is largely determined by the cutoff frequency of DCT basis function, and it directly affects the accuracy and runtime of NLR, which is defined by the following equation:
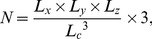
(8)where *L_x_*, *L_y_*, and *L_z_* are the Euclidean lengths of the reference image in the *x*-axis, *y*-axis, and *z*-axis, respectively, and *L_c_* (measured in millimeters (mm) determines the cutoff frequency. In the offline spatial normalization of SPM, the default *L_c_* of 25 mm, which is also sufficient for the structural MRI spatial normalization, makes the runtime much longer than a TR. Thus, considering fMRI images with a relatively lower spatial resolution than the structural MRI image, appropriately increasing *L_c_* is reasonable and necessary for the online process.

Using the bisection method, the value of *N* with a default *L_c_* of 25 mm is reduced by half of the previous value over three instances. Determined by Equation (8), the corresponding *L_c_*s are 31, 40, and 50 mm. The appropriate range of *L_c_* is discussed to balance the runtime and accuracy of spatial normalization, where the accuracy is described using the mean squared error (MSE) [37], [38].

### Assessment Scheme of Online Spatial Normalization

**Figure 2:**
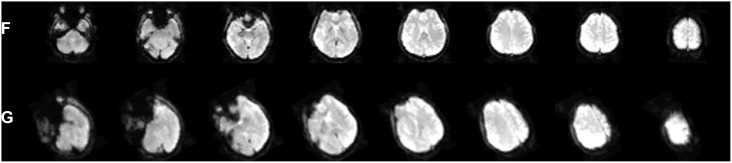
Construction of simulation data. The source image (**F**) was the first image in the real data, which was zoomed 0.7 times in advance. The reference image (**G**) was the source image transformed using the given parameters (Table 1).

The assessment consisted of two steps and was performed on a personal computer with an Intel Core CPU (Intel(R) Core(TM) i7-3770 CPU @ 3.4 GHz). First, compared with traditional AFR, the accuracy and runtime of the proposed PA-GN(β) AFR were validated in both simulation and real data. As described in Figure 2, the simulation data were constructed based on each subject’s first image using the given parameters in Table 1 to assess the accuracy of AFR. The maximum distance D_max(1,2)_, the matrix correlation coefficient between two transformation matrices **M**_1_ and **M**_2_, and MSE were used to compare the difference between the traditional AFR and PA-GN(β) AFR. The D_max(1,2)_ was defined as follows:
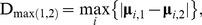
(9)where the coordinates **µ** *_i,_*_1_, **µ** *_i,_*_2_ were the resampled positions of voxel *i* in the reference image transformed by **M**_1_ and **M**_2_, respectively. In addition, the convergence rate and iteration number were used to further assess the performance of PA-GN(β) AFR.

**Table 1:** Estimated affine parameters, differences between the two transformation matrices, MSE and runtime using two AFR methods in simulation data.

		Given Parameters	Traditional AFR	PA-GN(β) AFR
**Translation/mm**	x	10	10.0223107±0.0179137	10.0223111±0.0179141
	y	−12	−12.0034754±0.0124250	−12.0034757±0.0124249
	z	−15	−15.0874369±0.0374636	−15.0874362±0.0374632
**Rotation/°**	x	10	10.0064315±0.0184493	10.0064331±0.0184481
	y	−20	−20.0298397±0.0251481	−20.0298423±0.0251474
	z	30	29.9833938±0.0308602	29.9833950±0.0308604
**Zoom**	x	1.1	1.0995272±0.0001853	1.0995272±0.0001853
	y	1.2	1.1985918±0.0003134	1.1985918±0.0003134
	z	0.9	0.9039135±0.0005948	0.9039135±0.0005948
**Shear**	x	−0.01	−0.0097952±0.0003445	−0.0097952±0.0003445
	y	−0.02	−0.0188380±0.0005639	−0.0188380±0.0005639
	z	0.03	0.0293605±0.0003499	0.0293604±0.0003499
**The maximum distance** D_max(r,e)_/mm			0.1547590±0.0204909	0.1547565±0.0204910
**The matrix correlation coefficient**			0.9999961±0.0000026	0.9999961±0.0000026
**Mean squared error (MSE)**			0.0090348±0.0013417	0.0090348±0.0013418
**Runtime (sec.)**			0.4337496±0.0381319	0.3318283±0.0153614

Second, after integrating PA-GN(β) AFR and NLR with an appropriate *L_c_*, the accuracy of online spatial normalization was assessed by brain activation in a finger-tapping task, compared with the offline spatial normalization derived from SPM8. The individual activation maps in the MNI space were obtained using online and offline spatial normalizations, respectively; other processes included realign, smooth using a Gaussian kernel in which the full width at half maximum (FWHM) was 8 mm, and general linear model (GLM) analysis. Both AFR and NLR of spatial normalization were only performed for the first image in real data. Next, using the estimated parameters, the image transformation was performed to normalize the subsequent images in this run, which had been realigned to the first image prior to spatial normalization. To quantify the difference in the activation maps made using the online and offline spatial normalizations, the activation coverage rate and the activation center distance of the whole brain and the defined ROIs were summarized. The activation coverage rate η was defined as follows:
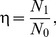
(10)where *N*_0_ was the number of activation voxels using offline spatial normalization and *N*_1_ was the number of co-activation voxels by online and offline spatial normalization. The activation center **A** was defined as (similar to the definition of centroid in Equation (2)).
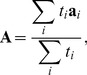
(11)where **a** *_i_* was the Euclidean coordinate of activation voxel *i* and *t_i_* was the *t*-value of the activation voxel *i*. The activation center distance was the Euclidean distance (mm) between the activation center following either online or offline spatial normalization.

## Results

### Comparison of Traditional AFR and PA-GN(β) AFR in Simulation Data

The AFR parameters estimated using traditional AFR and PA-GN(β) AFR, which fitted the image **F** to image **G** (Figure 2), were both similar to the given parameters; the difference between each pair was less than 10^−5^ (Table 1). The maximum distance (D_max(r,e)_) between the referenced matrix **(M**_r_), which consisted of the given parameters, and the matrix (**M**_e_), which consisted of the parameters estimated using traditional AFR or PA-GN(β) AFR, was much less than a voxel size (3.1×3.1×4.8 mm^3^) for both AFR methods. In addition, the correlation coefficients of **M**_r_ with **M**_e_ derived from the two AFR methods were both almost 1 (Table 1). A paired *t*-test between the MSE of traditional AFR and PA-GN(β) AFR across subjects showed that there was no significant difference (*p* = 0.9999).

**Figure 3:**
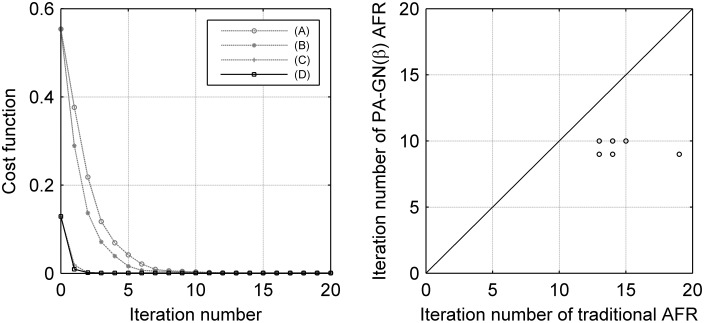
Simulation: convergence condition (left) and the required iteration number (right). (A) AFR without improvements; (B) AFR with β parameter only; (C) AFR with PA only; (D) PA-GN(β) AFR. The mean iteration number using the traditional AFR was 14.05±1.24, while that using the PA-GN(β) AFR was 9.50±0.50¸ which is approximately two-thirds of the traditional AFR requirement. Each dot represented a subject in the right figure, and there were fourteen dots overlapping with the six dots, as shown.

The mean cost function change with the number of iterations across subjects showed the convergence rate of the optimization process using GN with or without different modifications (Figure 3, left). The β parameter induced a faster GN convergence, and the PA provided an initial estimate for GN that significantly reduced the cost function. The iteration numbers obtained using traditional AFR and PA-GN(β) AFR for each subject were compared (Figure 3, right) and showed that many fewer iterations were required for PA-GN(β) AFR compared with traditional AFR. Moreover, the runtime of PA-GN(β) AFR was 0.1 s less than that of traditional AFR (Table 1).

### Comparison of Traditional AFR and PA-GN(β) AFR in Real Data

**Figure 4:**
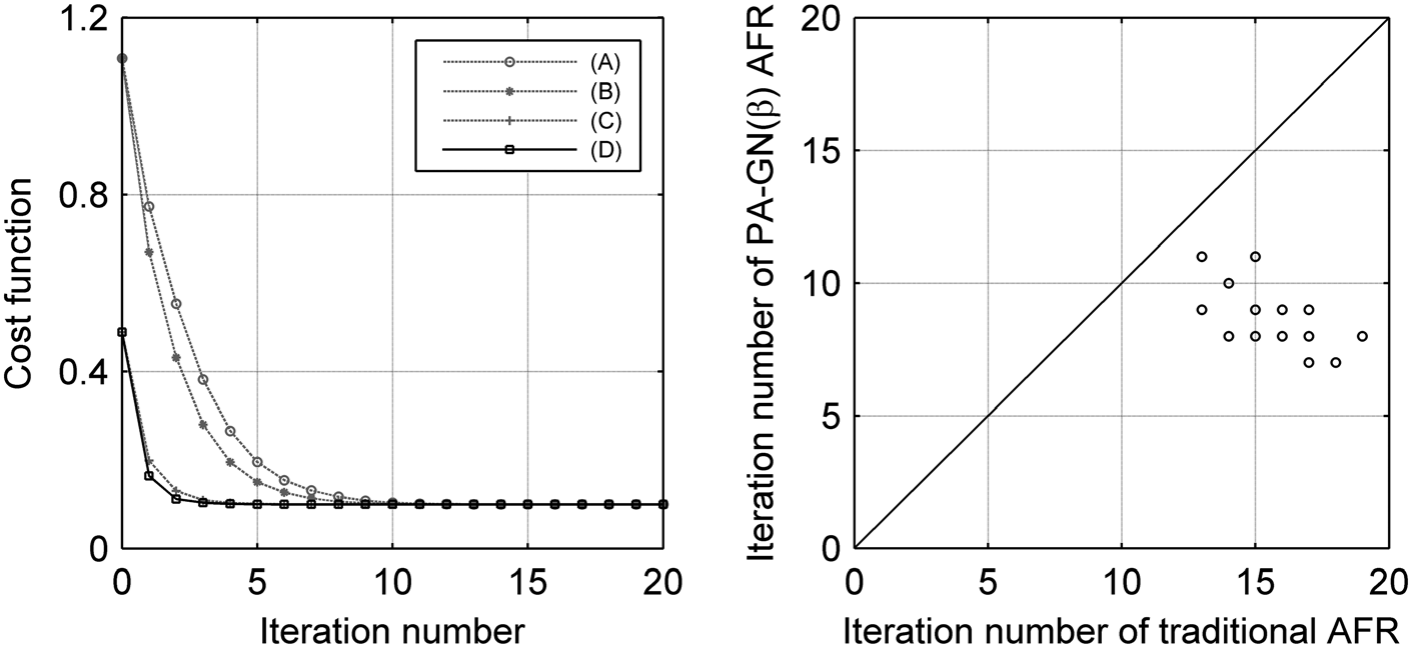
Real data: convergence condition (left) and the required iteration number (right). (A) AFR without improvements; (B) AFR with the β parameter only; (C) AFR with PA only; (D) PA-GN(β) AFR. The mean iteration number using the traditional AFR was 15.75±1.58, while that using the PA-GN(β) AFR was 8.60±1.07, which is approximately 50% of the traditional AFR requirement. Each dot represented a subject in the right figure, and there were six dots overlapping with the fourteen dots, as shown.

In real data, the first image was affine registered to the EPI template of SPM8, in which the AFR parameters were also estimated using both PA-GN(β) AFR and the traditional AFR. The maximum distance (D_max_) between the matrices estimated using traditional AFR and PA-GN(β) AFR was 0.1317±0.2293 mm, which was much less than one voxel size, while the matrix correlation coefficient of the two matrices was 0.99995±0.00014. Before AFR, the MSE between the first image of real data and the EPI template was 0.7301±0.0701. After traditional AFR, the MSE was reduced to 0.2851±0.0148, and after PA-GN(β) AFR, the MSE was reduced to 0.2854±0.0152. A paired *t*-test between the MSE of traditional AFR and PA-GN(β) AFR across subjects showed that there was no significant difference (*p* = 0.9582). In real data, the results of the convergence rate and the iteration number were similar to the findings in the simulation data (Figure 4); the runtime of PA-GN(β) AFR was 0.3285±0.0349 s, which was 0.2 s less than that of traditional AFR (0.5187±0.0498 s).

### Integration of PA-GN(β) AFR and NLR with Different Cutoff Frequencies

After PA-GN(β) AFR, NLR with different *L_c_*s was adopted in real data to complete the online spatial normalization. The MSEs were compared with those obtained using offline spatial normalization via a paired *t*-test across subjects (Table 2). With an *L_c_* of 50 mm, the MSE was significantly increased (*p* = 0.0497), whereas there was no significant difference in MSE when *L_c_* was 40 mm (*p*>0.1). Thus, 40 mm was considered the maximum *L_c_*. The runtime was also summarized in Table 2. With an *L_c_* of 25 mm, the total runtime was more than one TR (2 s), while the runtime with an *L_c_* of 31 mm was less than one TR but was still slow over the entire rtfMRI process. Thus, 31 mm was considered the minimum *L_c_*; *L_c_* for online spatial normalization was in the 31–40 mm range, and 35 mm was selected as the typical *L_c_* value that could maintain a balance between accuracy and runtime (Table 2).

**Table 2:** Accurary and runtime of online spatial normalization with different cutoff frequencies.

L_c_/mm	Accurary	Runtime (sec.)		
	MSE*	Image registration	Image transformation	Total
**25**	0.2626±0.0135	2.0520±0.2475	0.0827±0.0083	2.1347±0.2453
**31**	0.2641±0.0135	1.2733±0.1126	0.0801±0.0066	1.3534±0.1149
**35**	0.2656±0.0136	1.0376±0.0860	0.0794±0.0064	1.1171±0.0850
**40**	0.2667±0.0138	1.0046±0.1038	0.0803±0.0067	1.0849±0.1040
**50**	0.2716±0.0143	0.8598±0.0705	0.0789±0.0054	0.9387±0.0734

*The MSE using offline spatial normalization with default *L_c_* of 25 mm was 0.2624±0.0135.

### Validation of Online Spatial Normalization via Brain Activation

**Figure 5:**
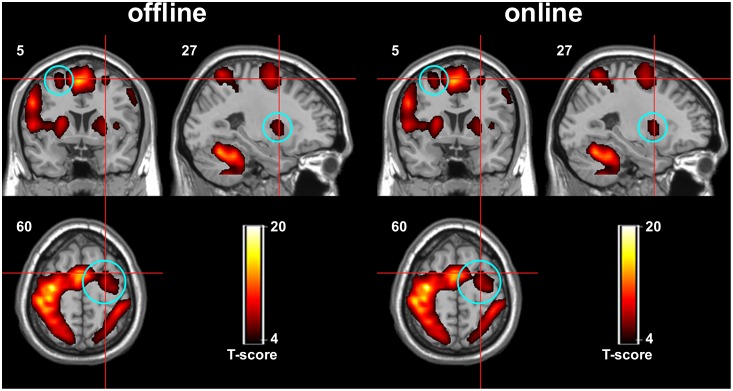
The group activation maps using offline spatial normalization (left) and online spatial normalization (right). There were slight differences, such as the areas indicated in the blue circles, but no significant differences were observed between the activation patterns.

Comparison of the individual activation maps using online and offline spatial normalization showed no significant differences across subjects (paired *t*-test, FWE (family wise error) correction, *p*>0.05). Furthermore, group activation maps obtained using a one-sample *t*-test (*p*<0.001, cluster size >20) showed that the supplementary motor area (SMA), premotor cortex (PMA), primary motor cortex (M1) and cerebellum (Cere) were all activated in the finger-tapping task using different spatial normalization methods (Figure 5). Six regions defined in the AAL atlas as the regions of interest (ROIs) were selected using the WFU_PickAtlas tool [39], and the activation coverage rate and activation center distance of each ROI were summarized (Table 3). The mean activation coverage rates of the whole brain and the defined ROIs were all greater than 90%, and most of the subjects’ coverage rates were greater than 90%. The activation center distances were all within the size of the resampled voxel (3×3×4 mm^3^) and were predominantly less than 2 mm.

**Table 3:** Activation coverage rate and activation center distance in different brain regions.

ROI	AAL Atlas	Activation Coverage Rate		Activation Center Distance	
		Rate (%)	Subject Number	Distance/mm	Subject Number
**Whole Brain**	**–**	94.56±4.51	18/20	0.6986±0.6425	18/20
**SMA**	**Supp_Motor_Area**	95.94±4.05	18/20	0.4884±0.2586	20/20
**Left PMA**	**Precentral_L**	96.85±3.04	18/20	0.4554±0.3155	20/20
**Right PMA**	**Precentral_R**	91.95±9.22	16/20	0.7167±0.3644	20/20
**Left M1**	**Postcentral_L**	97.67±1.36	20/20	0.3660±0.3238	20/20
**Right M1**	**Postcentral_R**	95.88±4.84	18/20	0.6867±0.5081	20/20
**Right Cere**	**Cerebelum_6_R**	96.86±3.79	19/20	0.1821±0.1705	20/20

## Discussion

The proposed online spatial normalization significantly improved the performance of traditional AFR using PA and the self-adaptive β parameter, while the accuracy was maintained the same as with the traditional AFR. In addition, the proposed method provided a reasonable way to determine the appropriate cutoff frequency of the DCT basis function in NLR. Overall, the proposed method completed within one TR and reached the runtime requirement of rtfMRI on the current typical personal computer, while its accuracy was relatively close to the offline spatial normalization method.

The PA, which has coarse but global properties, is a rigid registration method based on the shape and intensity of the entire brain image. Without a suitable initial estimate, the GN method would require a large number of iterations and might not reach the global optimum [40]. It has been shown that the initial estimate provided by PA could significantly decrease the iteration number of GN, and it can be inferred the PA might have the potential to reduce the probability that GN cannot find the global optimum. However, there are some practical issues in PA. First, if the scan orientation of the source image is not identical to the orientation of the reference image (e.g., a sagittally scanned source image and an axially scanned reference image), the estimate obtained using PA will be incorrect [31]. To eliminate the constraint on scan orientation, higher moments can be introduced into the PA [41], which would cause extra computational loads. Second, the PA requires whole brain information; if the brain is partially scanned, the estimate by PA could deviate. The whole brain scan is required by this method in practice.

The self-adaptive β parameter can improve the performance of GN while maintaining stability and accuracy. The β parameter, which is dependent on the change rate of cost function, could achieve the same effect as the α parameter [35] on the self-adaptive adjustment of the iteration step using the *λ* constraint factor to limit the adjustment extent. Moreover, the definition of *λ* is related to the complexity of the registration method. For example, *λ* should be smaller in a rigid registration.

**Figure 6:**
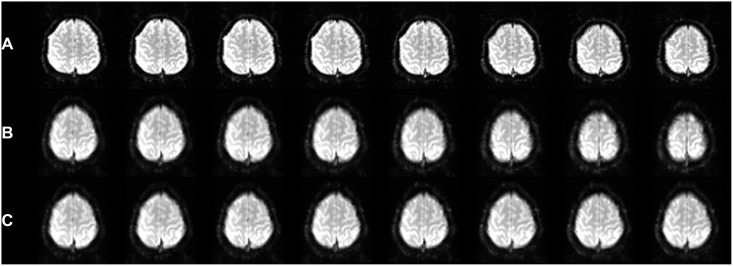
The negative effects of inter-run motion accumulation avoided using online spatial normalization. The images were obtained from one subject’s first image in each of eight on-going runs of an rtfMRI experiment, which lasted approximately 90 mins. **A.** The same slices of source images in different runs showed that the inter-run motion accumulated, and the image in the last run was different from the image in the first run by almost one slice. **B.** The corresponding slices in the normalized images using offline spatial normalization. The normalization parameters were estimated only in the first run, which simulated the processes of the previous spatial normalization methods [25], [26], [42]. It was evident that the inter-run motion negatively affected the accuracy of the normalized images in the subsequent runs. **C.** The corresponding slices in the normalized images by online spatial normalization using the parameters estimated in each run. The inter-run motion was effectively avoided, and the normalized images were nearly the same.

As a real-time algorithm in rtfMRI, the proposed method can also avoid the negative effect of inter-run motion on the accuracy of the normalized images and ROI position. During the rtfMRI experiment, which commonly consists of a series of runs and may last a long time, it is impossible for the subject to keep his head immobile; the inter-run motion thus accumulates and sometimes cannot be ignored in the continuous runs (Figure 6A). The accuracy of the normalized images using previous methods [25], [26], [42] can be reduced by the inter-run motions along with on-going runs (Figure 6B) because the normalization parameters estimated in the previous run could be imprecise for the moved brain images acquired in the following online runs. Furthermore, in most rtfMRI experiments [20], [21], [43], [44], the ROI spatial position selected in only one previous run could also be biased by the inter-run motion. The proposed online spatial normalization can be performed in each run; it has the benefit of avoiding these limitations (Figure 6C).

Online spatial normalization can be valuable for investigations and applications of rtfMRI, but some details need to be specified and improved when applying it in rtfMRI. In each run, the images are normally aligned to the first image by head motion correction before the normalization, which makes the rigid differences between these realigned images relatively small. Thus, both AFR and NLR can be only performed for the first image in each run. Then using the estimated parameters, the image transformation is performed to normalize the subsequent images in the same run, which have been realigned to the first image prior to spatial normalization. In addition, considering the DCT basis functions are lack of physical meaning with respect to inter-subject anatomical variability, other alternative NLR method with lower computational loads could be used for online spatial normalization. The improvement on NLR methods needs further investigations.
